# RNA Modulators of Complex Phenotypes in Mammalian Cells

**DOI:** 10.1371/journal.pone.0004758

**Published:** 2009-03-09

**Authors:** Angela Lai, Murray J. Cairns, Nham Tran, Hong-Ping Zhang, Lara Cullen, Greg M. Arndt

**Affiliations:** 1 Johnson and Johnson Research Pty Ltd, Australian Technology Park, Eveleigh, New South Wales, Australia; 2 The Sydney Head and Neck Cancer Institute, Sydney Cancer Centre, Royal Prince Alfred Hospital and University of Sydney, Sydney, New South Wales, Australia; Victor Chang Cardiac Research Institute (VCCRI), Australia

## Abstract

RNA-mediated gene silencing, in the form of RNA interference (RNAi) or microRNAs (miRNAs) has provided novel tools for gene discovery and validation in mammalian cells. Here, we report on the construction and application of a random small RNA expression library for use in identifying small interfering RNA (siRNA) effectors that can modify complex cellular phenotypes in mammalian cells. The library is based in a retroviral vector and uses convergent promoters to produce unique small complementary RNAs. Using this library, we identify a range of small RNA-encoding gene inserts that overcome resistance to 5-fluorouracil (5-FU)- or tumour necrosis factor alpha (TNF-α)- induced cell death in colorectal cancer cells. We demonstrate the utility of this technology platform by identifying a key RNA effector, in the form of a siRNA, which overcomes cell death induced by the chemotherapeutic 5-FU. The technology described has the potential to identify both functional RNA modulators capable of altering physiological systems and the cellular target genes altered by these modulators.

## Introduction

The introduction of double-stranded RNA (dsRNA) into a range of organisms induces both a potent and specific gene silencing effect termed RNA interference (RNAi) [1]. These dsRNAs are processed by Dicer to produce 21–23 nucleotide duplex small interfering RNAs (siRNA) with 2 nucleotide 3′ OH overhangs that act as the effectors of gene silencing [2]. Furthermore, it has been demonstrated that chemically synthesised 21 bp siRNAs can be used to induce gene silencing in mammalian cells [3], [4]. The transient nature of the gene silencing effect invoked by siRNAs, and the prohibitively high costs of chemical synthesis, has led to the development of DNA vectors capable of expressing siRNAs intracellularly. Expression cassettes have been developed using the endogenous U6 small nuclear RNA (snRNA) or H1 RNA polymerase III promoters to drive expression of sequence-specific small hairpin RNAs (shRNAs) that stably regulate gene expression in mammalian cells via RNAi [5], [6]. As an alternative approach, some groups have used the co-expression of sense and antisense RNA strands from independent expression cassettes or a divergent promoter [7]. The use of convergent transcription from opposing promoters to induce RNAi-mediated gene inhibition has been reported in trypanosomes and Drosophila [8], [9]. More recently, convergent transcription-induced RNAi has been demonstrated to be an effective way of controlling specific gene expression in mammalian cells [10], [11].

RNAi is also a powerful genetic tool for loss-of-function studies. Large-scale RNAi libraries have been generated for use in forward genetics screens with the aim of linking genes with specific cellular phenotypes [12], [13]. Genome-wide RNAi-based genetic screens were first demonstrated in *C.elegans* and Drosophila cells [14], [15]. More recently, this genetic screening approach has been applied to mammalian cell systems using different forms of RNAi libraries. Initially, large-scale shRNA expression libraries were constructed using computational algorithms and “bar-coded” oligonucleotides [16], [17]. With a better understanding of the characteristics associated with natural miRNA processing and to improve the efficiency of shRNA activity, second generation genome-wide shRNA libraries were produced using pre-miRNA flanking sequences [18]. The above libraries have been used for loss-of-function studies in mammalian cell culture and to identify pre-selected genes contributing to complex cellular phenotypes. In an alternative strategy for producing targeted shRNA libraries, several groups have developed enzymatic methods for converting cDNAs into shRNAs for use in genetic screening [19]–[21]. All of the aforementioned libraries target specific protein coding genes or restricted cDNA sub-populations and are particularly useful for identifying single gene targets involved in a biological or disease process. These libraries do not necessarily permit the identification of completely novel small RNAs or targets involved in complex cellular physiology.

In addition to the use of shRNAs several groups have reported on the use of convergent transcription to direct RNAi-mediated silencing of specific genes [10], [11], [22]. In this strategy two opposing RNA polymerase II or III promoters are used to drive transcription of sense and antisense RNAs from a single DNA insert. The complementary RNAs then form a duplex RNA which is processed by Dicer and loaded into the RNA induced silencing complex (RISC). Different forms of convergent RNAi libraries have been produced and used in genetic selections [22]–[24]. However, as with the majority of genome-wide shRNA libraries, these libraries are restricted to surveying previously reported target genes.

One alternative to the production of RNAi expression libraries using known genes involves the generation of random libraries capable of expressing a universal set of shRNAs or siRNAs. Several enzymatic methods have been described for the production of shRNA expression libraries using degenerate oligonucleotides [25]–[27]. Most recently, a random shRNA-encoding retroviral library was used to identify shRNA sequences that double the survival of mouse pro-B cells following IL-3 withdrawal [28]. Random mutagenesis and re-screening were then used to further optimize the shRNA hits toward the development of novel therapeutics. Convergent promoter-based random siRNA expression libraries have been produced by cloning random 19-mer DNA sequences between two opposing RNA polymerase III promoters in plasmid vectors [29]. This library was used for phenotype-driven screening to identify siRNAs inducing cell growth. The advantages associated with using a convergent promoter format for producing random RNAi libraries are the potential for producing small RNAs that operate by canonical and non-conventional mechanisms to control single or multiple target genes and the possibility of discovering novel, multifunctional small RNAs capable of modifying complex cellular phenotypes. These small RNAs have the potential to be novel therapeutics or biological tools. Unfortunately, there have been few reports of the use convergent random RNAi libraries for genetic screening in mammalian cells and these have been restricted to the use of plasmid-based libraries.

Here, we report on the development and application of a convergent random small RNA expression library for use in mammalian cell types to isolate novel RNAs capable of modifying complex cellular phenotypes. This retrovirally-based expression library was used in two independent unbiased genetic selection assays aimed at overcoming cell death induced by the chemotherapeutic 5-FU or the cytokine TNF-α. Using this approach, novel small RNA effectors were identified that alter these phenotypes. Further analysis demonstrated that one of these effectors operated through the canonical RNAi pathway. We discuss the utility of this technology for finding new gene functions through phenotypic selection and discovering novel small RNAs that can be used to manipulate complex cellular phenotypes associated with human disease states.

## Results

### Production of retroviral vector with convergent promoters

In a previous study we reported on the use of convergent transcription from two U6 RNA polymerase III promoters in a plasmid-based system to induce RNAi-mediated gene silencing in mammalian cells [10]. The suppression of target gene expression was dependent on Dicer and the convergent promoter cassette operated in both transiently and stably transfected cell populations. To develop a random small RNA expression library suitable for large-scale genetic selections, we constructed a modified pLXSN retroviral vector containing the human U6 and H1 RNA polymerase III promoters in opposite orientation to each other (designated pHybrid). Retroviral vectors have been successfully used for both biased and unbiased genetic screens in mammalian cells and provide an optimal vehicle for delivery of highly complex genetic libraries [12].

**Figure 1:**
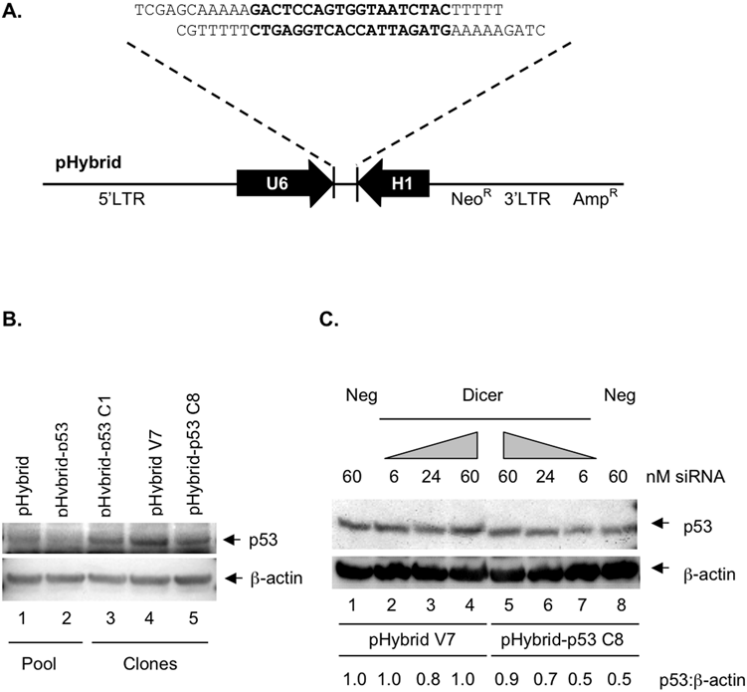
RNAi-mediated suppression of p53 using convergent transcription within the retroviral vector pHybrid-p53. (A) Schematic of the core pHybrid-p53 vector including the p53-specific insert sequence encoding sense and antisense RNAs. The core pHybrid vector contains the human U6 and H1 promoters in opposing orientation. (B) Suppression of p53 protein levels in HCT116 pooled populations and clones stably transduced with pHybrid-p53. HCT116 cells transduced with pHybrid-p53 were selected in G418 for eight days to produce stable pools. These pooled populations were serially diluted to isolate stable clones (indicated by V7 for pHybrid alone and C1 and C8 for pHybrid-p53). Total protein lysates were assessed for levels of p53 and β-actin, the latter as the loading control. (C) Dicer-dependent control of convergent transcription-induced suppression of p53. HCT116 stable clones containing pHybrid (V7) or pHybrid-p53 (C8) were transiently transfected with different concentrations of the Dicer-specific siRNA (Dicer) or a single concentration of the negative control siRNA (Neg). Levels of p53 and β-actin were determined from isolated protein lysates.

To determine whether the convergent U6/H1 expression cassette could produce functional siRNA that mediated effective gene suppression, an insert encoding a p53-specific siRNA [6] was enzymatically-generated (as described in materials and methods) and cloned into pHybrid (Figure 1A). The pHybrid-p53 retroviral vector was transduced into HCT116 cells and Western analysis performed on both pooled populations and single clone isolates to determine the effect of siRNA expression on p53 protein levels. As shown in figure 1B, convergent transcription of the p53-specific insert resulted in a 50% reduction in p53 protein levels within both the pooled population (Figure 1B, lane 2), and two clonal lines (Figure 1B, lanes 3 and 5), when compared to vector controls (Figure 1B, lanes 1 and 4, respectively). We also examined the responsiveness of these clones to 5-FU treatment and showed that cells containing the pHybrid-p53 construct displayed an increased proliferative potential and reduced apoptosis (data not shown), a phenotypic profile consistent with resistance to 5-FU treatment.

Given the use of a convergent promoter cassette, and the expression of two complementary RNAs with the potential to form dsRNA, there were several possible ways by which the RNA effector encoded in pHybrid-p53 could direct suppression of p53 gene expression. To examine the mechanism of p53 suppression, we attenuated the levels of Dicer, a key enzyme involved in the processing of dsRNA to produce effective siRNA, by using a synthetic siRNA targeted against Dicer mRNA [30]. Delivery of Dicer-specific siRNA to cells stably containing pHybrid-p53 restored p53 protein levels to basal levels in a dose-dependent manner (Figure 1C, lanes 5–7). In contrast, the same cells transfected with a negative control siRNA, having no known target within mammalian cells, displayed the same 50% reduction in p53 protein (Figure 1C, lane 8). Furthermore, there was no modulation of p53 protein in vector control cells transfected with the same dose of siRNA specific for Dicer (Figure 1C, lanes 2–4). These results indicate that the convergent transcription of the p53-specific insert in pHybrid-p53 produced siRNA that was effective at reducing p53 protein levels and this suppression was Dicer-dependent.

### Generation of a randomized small RNA retroviral expression library

**Figure 2:**
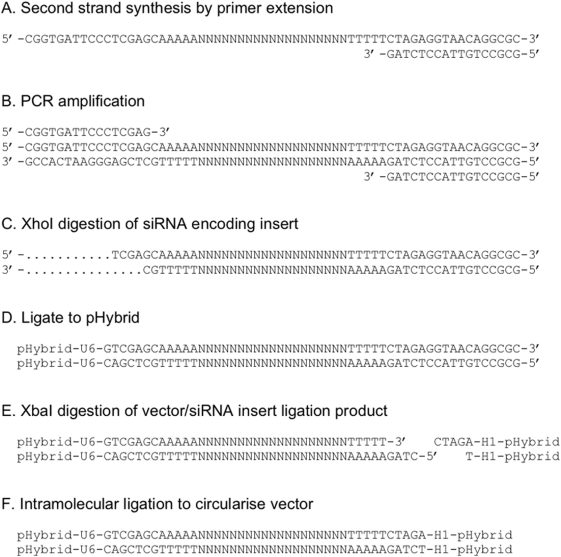
Enzymatic synthesis of inserts for construction of the random small RNA expression library. A 63 base oligonucleotide, composed of 19 random nucleotides (N) flanked by five adenosines and five thymidines, XhoI and XbaI restriction sites and primer binding sites, was extended to produce a second strand using the indicated primer (A). This dsDNA template was amplified with PCR primers (B) and subject to XhoI digestion (C). Following ligation to the SalI-digested pHybrid (D) and isolation of ligated products, the mix was digested with XbaI (E) and subjected to intramolecular ligation to produce the final random siRNA library (F).

Following the confirmation of the activity of the U6 and H1 promoters within the context of the retroviral vector backbone, we constructed and characterised an expression library in pHybrid containing random insert sequences. The starting synthetic oligonucleotide was composed of 19 nucleotides of randomised sequence flanked on the 5′ end with a primer-binding site, an XhoI site and five adenosines. On the 3′ side of the randomised nucleotides was a string of five thymidines followed by an XbaI site and a second primer-binding site. Using the enzymatic method described in Figure 2, a pool of randomised double-stranded DNA inserts was produced and cloned into pHybrid. To reduce the presence of concatemers, and increase the proportion of library members with single inserts, we used sequential end ligation [31]. In this process, the dsDNA oligonucleotides were ligated in excess to a de-phosphorylated plasmid using one compatible overhang, resulting in a pool of linear plasmids containing insert(s) in either orientation. Further enzymatic digestion to generate the second compatible overhang allows intramolecular ligation and plasmid circularisation. Using this method, we produced a random small RNA expression library composed of approximately half a million clones. Characterisation of this library indicated that most inserts were unique and aligned to different regions of the human genome. Although the complexity of the library was rather low, the use of convergent promoters provided the potential to encode small antisense RNAs, sense RNAs, dsRNAs or microRNA-like RNAs from a single library plasmid member.

### RNA effector identification using the small RNA expression library

**Figure 3:**
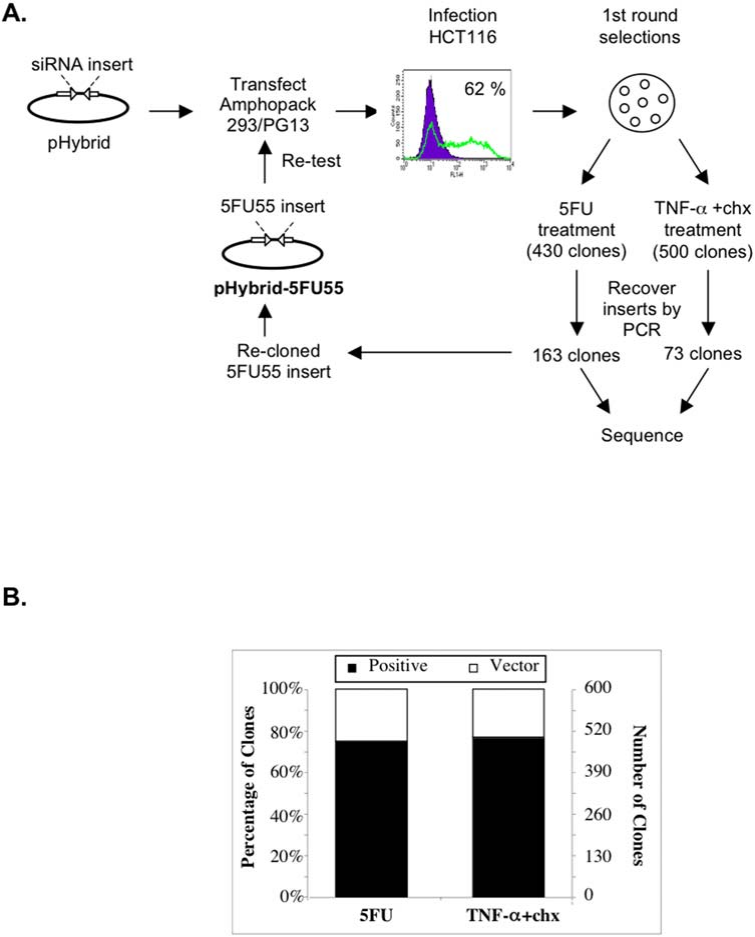
Overview of the cell-based genetic selection assays performed using the random small RNA expression library. (A) Plasmid DNA from the random siRNA library was transfected into a mixture of Amphopack HEK293 and PG13 packaging cells. The viral containing medium was used to infect 62% of HCT116 cells. Following selection in G418, stably trasnduced cells were subjected to two independent genetic selections involving treatment with 5FU or TNF-α plus cycloheximide (chx). At 14 days after removal of the selective agent, independent surviving colonies were isolated and subjected to PCR to recover resident library vector inserts. These sequences were used for target identification and insert 5FU55 was re-cloned into pHybrid for re-delivery and validation. (B) Percentage and number of clones recovered from the two genetic selections. The filled portion of the histograms represents the numbers associated with the library-containing population (positive) and the open portion indicates the vector alone (vector).

The random small RNA library was used in two independent functional screens to identify RNA effectors conferring resistance to 5-FU- or TNF-α-induced apoptosis. This library was delivered to HCT116 cells and a pooled population generated following eight days of selection in the presence of G418. These cells were subjected to either 400 uM 5-FU treatment for 18 h or 25 ng/ml TNF-α and 50 ug/ml cycloheximide co-treatment for 72 h, conditions that were previously determined to induce cellular apoptosis in HCT116 cells (data not shown). The surviving resistant clones were isolated, genomic DNA extracted, library inserts recovered by PCR and insert sequences identified by sequence analyses (Figure 3A). A total of 430 and 500 resistant clones were recovered with 5-FU treatment and TNF-α plus cycloheximide co-treatment, respectively. The number of background colonies identified using the same conditions on vector alone cells was only 20–25% of the number obtained with the library-containing cells (Figure 3B). This suggested that library constituents existed that potentially overcame 5-FU or TNF-α-induced cell death.

**Figure 4:**
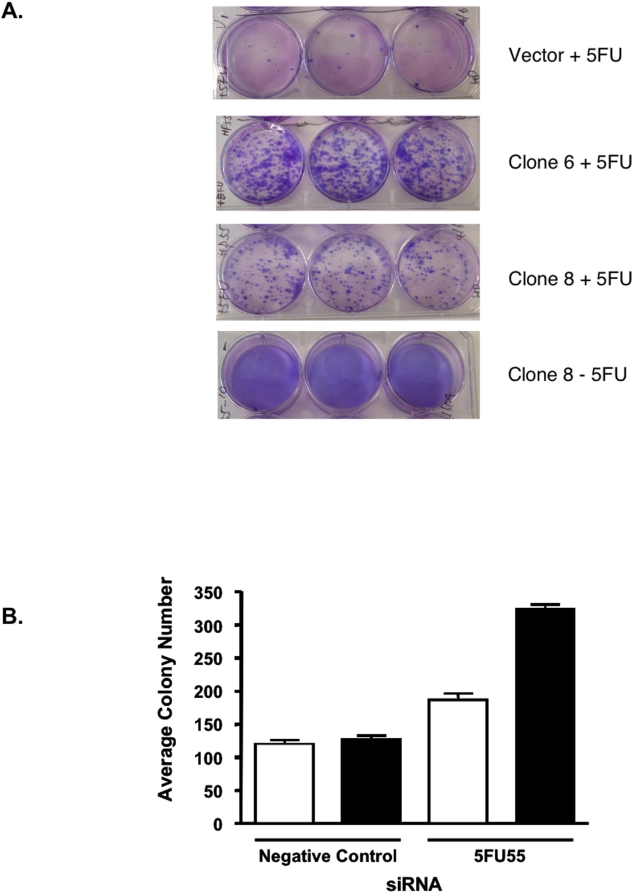
Resistance to 5FU conferred by 5FU55. (A) Re-delivery and validation of pHybrid-5FU55 as conferring resistance to 5FU. The original 5FU55 insert was subcloned into pHybrid and re-delivered to HCT116 cells via retroviral transduction. Independent clones were isolated, exposed to 5FU for 48 h and then assessed for colony numbers at 14 days. Panel 1: pHybrid vector alone treated with 5FU. Panel 2: clone 6 containing the pHybrid-5FU55 vector and treated with 5FU. Panel 3: clone 8 containing the pHybrid-5FU55 vector and treated with 5FU. Panel 4: clone 8 containing the pHybrid-5FU55 vector without prior exposure to 5FU. (B) Transient transfection with 5FU55 siRNA produces increased resistance to 5FU. HCT116 cells were transfected with two different concentrations (open histograms = 30 nM; filled histograms = 60 nM) of either negative control siRNA or 5FU55 siRNA. After 48 h, cells were exposed to 5FU for 18 h and then re-seeded. At 14 days, colonies were stained with crystal violet and counted.

From the 5-FU and TNF-α genetic selections, the library insert sequences from a total of 163 and 73 independently-derived HCT116 resistant clones were determined, respectively. Of particular interest was the enrichment of a single insert sequence in 55 independent 5-FU resistant clones of the 163 clones analysed. This insert sequence (designated 5FU55) was not represented in the any of 384 sequences of the library prior to selection or in any of the sequences identified among the clones resistant to TNF-α-induced cell death. To validate this insert sequence as conferring resistance to 5-FU, the 5FU55 DNA was recovered by PCR from a 5-FU resistant clone in the original screen, re-cloned into pHybrid to produce pHybrid-5FU55 and re-delivered into HCT116 cells via retroviral transduction. Single clones containing either the vector alone or pHybrid-5FU55 were obtained following selection and characterised for resistance to 5-FU using a clonogenic assay. All clones receiving pHybrid-5FU55 formed colonies following exposure to 5-FU, with cellular resistance ranging from intermediate to robust (compare clones 8 and 6, respectively, in figure 4A). In contrast, HCT116 cells transduced with the vector alone displayed only background colony formation (Figure 4A). All clones receiving either pHybrid or pHybrid-5FU55 showed similar growth profiles in the absence of 5-FU treatment, suggesting that the small RNA effector expressed from the 5FU55 DNA did not alter general cell physiology.

### Synthetic 5FU55 siRNA

To further define the RNA effector encoded by the 5FU55 insert, and determine whether small RNAs identified using the random small RNA library could be converted to synthetic RNA effectors, we designed and tested a synthetic siRNA corresponding to the unique sequence contained in the 5FU55 insert. HCT116 cells were transiently transfected with the 5FU55-siRNA and cells exposed to 400 uM 5-FU and examined for clone formation using a clonogenic assay. In comparison to HCT116 cells transfected with the negative control siRNA, cells receiving the 5FU55 siRNA displayed a dose-dependent increase in colony formation following exposure to 5-FU (Figure 4B). This suggests that the insert contained within the original random library insert encoded for a siRNA capable of modifying the chemosensitivity phenotype of HCT116 cells. Interestingly, simple sequence alignment analysis using the passenger or guide RNA strands of 5FU55 and the publicly available human RefSeq database did not identify a single target transcript with complete complementarity to these small RNA sequences. Instead, using the criteria of at least 14 bases of contiguous complementarity with the 5′ end of the guide strand, we identified 14 different target mRNAs with the potential to form a partial duplex. The targets identified included the homeodomain-interacting protein kinase-2 (HIPK2), tektin 1, a lipid raft linker protein (RFTN1), and a member of the keratin protein family (keratin 73). These results suggest that the 5FU55 siRNA conferred resistance to 5FU by directly or indirectly modulating the expression levels of more than one target gene product.

## Discussion

In this paper we describe the construction and characterisation of a random small RNA expression library and demonstrate the utility of this library for identifying small RNA effectors that alter complex cellular phenotypes. Using two different genetic selection assays, we identified constituents within the library that encode small RNAs capable of overcoming both 5-FU and TNF-α-induced apoptosis. Following one round of genetic selection for resistance to 5-FU, a single insert was enriched and contained within 30% of the 5-FU-resistant clones. The link between this library insert and the 5-FU resistant phenotype was confirmed by re-delivering the specific vector containing this insert. Moreover, the novel sequence contained within the insert was used to both identify potential target proteins directly or indirectly suppressed by the expressed RNA effector and to produce a synthetic siRNA capable of producing the same modified protein expression profile and altered complex cellular phenotype.

Most of the gene-expressed RNAi libraries used for target identification and validation have been produced using computer algorithms and previously known genes or gene families. These designs have incorporated single promoters controlling shRNAs [16], [17] or dual opposing promoters driving expression of complementary sense and antisense RNAs [22], and required computational prediction of accessible target sites. One alternative that complements these targeted libraries, and eliminates the need for computational prediction and experimental confirmation of effective RNAi effectors, is the use of RNAi expression libraries containing randomised inserts. In this study we report on the construction, characterisation and application of a random RNAi library using convergent RNA polymerase III promoters based in a retroviral vector. There are several advantages associated with this form of RNAi expression library. Firstly, the opposing promoter arrangement leads to the production of two complementary RNAs that have the potential to form not only dsRNA and mediate RNAi-directed gene silencing, but also small RNA effectors that can operate through other pathways. These include single stranded antisense RNA, sense RNA, or miRNAs [32]. This expands the kind of RNA effectors and therefore the intracellular pathways that can be used for silencing gene expression. Secondly, the library reported in this study is replete of repeat sequences thus eliminating instability. Thirdly, the RNA polymerase III promoters can be used in different cellular systems, thus expanding the applicability of the library. Fourthly, a random library is not restricted to known genes or protein coding genes as targets. It can encode small RNA effectors capable of targeting ncRNAs, the latter of which have been shown to contribute to complex disease-associated phenotypes [33]. Finally, this form of RNAi library is cost-effective and amenable to use in both small and large laboratories for gene discovery and identification of small RNA effectors, especially when used in combination with functional genetic screens or selections.

One of the criticisms associated with the use of randomized RNAi expression libraries is the limited number of representative inserts that can be incorporated into an expression vector. For example, it is not possible to construct and screen all insert sequences of 19 bases as this would require a library of 2.7×10^11^ different constructs. However, it has been well reported that small RNAs can be effective against single and multiple target mRNAs with incomplete complementarity [34], [35]. Studies with synthetic siRNAs have identified the limited requirements for an effective guide strand [36]–[38]. Mutations within termini of siRNA do not significantly impact silencing [39]. Moreover, mRNAs with as few as 11 nucleotides of homology can be regulated by a siRNA [34]. Even if mismatches within the body of a siRNA reduce or eliminate its ability to mediate target mRNA cleavage, it is possible that this duplex RNA could still operate as a miRNA-like translational inhibitor [36]. Furthermore, naturally occurring miRNAs only require matches within an eight base seed region to mediate control of up to 200 different protein coding targets [40]. The above indicates that convergent small RNA libraries have the potential to encode multiple effectors without the need for precise sequence complementarity.

By combining the random RNAi library with cell-based genetic selections, it is possible to identify RNA modulators of complex phenotypes both for use as biological tools and potential therapeutics. In this study we used the library in two functional genetic selection assays to identify small RNA effectors capable of overcoming cell death induced by a chemotherapeutic agent (5FU) or a cytokine (TNF-α). Unique insert sequences were identified within each genetic selection with few clones containing the same insert sequence. This is not surprising considering that most genetic selections require multiple rounds of re-selection to enrich for specific inserts [12]. There were no shared sequences between the two different genetic selections. This may reflect the different biological pathways used by 5-FU and TNF-α for mediating cell death. Furthermore, the sequences identified following genetic selection in mammalian cells were not present among the bacterial plasmids sequenced in the pre-selected library. Interestingly, even with a single round of genetic selection, we were able to identify a single insert enriched in a third of all 5FU resistant clones. More recently, we have expanded the utility of this expression library and identified ten small RNA effectors conferring resistance to cellular infection by the respiratory syncytial virus (Fred Delvecchio, unpublished results). Further analysis of the insert sequences identified through the different genetic selections did not reveal the presence of common “seed” regions or extended sequence similarity or complementarity with previously reported human miRNAs. The power of this approach is its unbiased nature that permits the cell itself to identify effective RNA agents that alter specific cellular phenotypes without directing cell toxicity. These RNA modulators have the potential to be used as gene-expressed or synthetic RNA leads for therapeutic development [28].

One common theme observed in using random RNAi expression libraries in unbiased genetic screens is the difficulty in translating the identified small RNA effector sequence into identification of the direct targets, the latter of which provide a better understanding of the underlying mechanism associated with modifying a complex disease-associated phenotype and specific candidates for conventional drug development [28]. In this study a simple sequence homology search indicated that the inserts identified through the two different selection assays did not recognise any sequences within the human transcriptome with complete complementarity and instead recognised between 7 and 20 different targets, using the criteria of 14 bases of contiguous homology. This suggested that all the effectors identified operate through control of multiple target genes to alter the phenotype under study, most probably through the promiscuous nature of the RNAi/miRNA machinery in mammalian cells [41], [42]. Given the random nature of the convergent RNAi expression library it was not surprising that it was difficult to identify precise targets of the effector RNAs. It is likely that these sequences do not exist in nature, may act through novel mechanisms and have both direct and indirect targets. Work with random shRNA libraries identified several shRNAs capable of overcoming IL3-dependent cell death; however these authors could not identify specific targets using simple homology searches or miRNA target prediction algorithms [28]. Further experimentation will be required in order to expand the random small RNA library approach to gene identification. Toward this end, the “rules” being identified for naturally-occurring miRNAs may provide an inroad to deciphering the molecular targets of the identified functional small RNAs.

In the case of the 5FU55 effector, we identified homeodomain-interacting protein kinase-2 (HIPK2) as one potential target. The development of resistance to 5FU is multifactorial and several different mechanisms have been reported [43]–[45]. These include loss of sensitivity to apoptosis, increased expression of DNA repair genes, changes in fluoropyrimidine metabolism, and altered drug transport. Our observation that HIPK2 is a potential target for the 5FU55 siRNA implicates one alternative pathway for invoking cellular resistance to 5FU. HIPK2 is a transcriptional co-repressor, and its suppression has been shown to inhibit chemotherapeutic drug-induced apoptosis [46]. For example, DNA damage induced by cisplatin or adriamycin activates HIPK2, leading to phosphorylation of p53 on serine 46 and activation of p53-mediated apoptosis [46]–[48]. A block in this cellular response pathway can lead to chemotherapeutic resistance. In the present study, inhibition of HIPK2 by the 5FU55 siRNA may have prevented transcriptional repression of the HIF-1 transcriptional activator. One result would be the induction of HIF-1 targets genes including the multidrug resistance gene MDR1 and the anti-apoptotic protein Bcl2 [49]. In addition, a reduced level of HIPK2 would impair activation of p53-directed apoptosis via DNA damage resulting from FdUTP misincorporation into DNA. The end result would be a multifactorial response leading to 5FU resistance. Interestingly, a subunit of HIF-1, HIF-1a, has been reported as being overexpressed in colorectal tumors and contributing to chemotherapeutic resistance [50]. The validity of these proposed mechanisms will require further experimentation.

The integrative approach using random small RNA libraries and cell-based genetic selection provides a novel technology platform for identifying RNA effectors capable of modifying complex cellular phenotypes through control of the expression level of multiple targets. These RNA effectors have the potential to be used as novel therapeutic candidates and tools for deciphering the underlying molecular pathways contributing to normal and disease-related phenotypes.

## Materials and Methods

### Cell culture

HCT116 colorectal cancer cells were cultured in McCoy's medium (Invitrogen) containing 10% FBS, penicillin, streptomycin, and glutamine. Amphopack HEK293 (Invitrogen) and PG13 packaging cell lines were maintained in Dulbecco's modified Eagle's medium (Invitrogen) containing 10% FBS, penicillin, streptomycin, glutamine and sodium pyruvate.

### Plasmid construction

To establish a vector system in which convergent promoters drive the expression of short complementary RNAs, we modified the pLXSN retroviral vector (Clontech) to include convergent human U6 and H1 RNA polymerase III promoters. The U6+1 promoter contained in pTZ(U6+1) (gift from David Engelke) was PCR-amplified using the following forward and reverse primers: 5′-GCGCCTCGAGATAGGGAATTCGAGCTCGGTA-3′ and 5′-GCGCGGATCCTTGTAAACGACGGCCAGTGC-3′. Following digestion with XhoI and BamHI, this DNA fragment was ligated into the multiple cloning site of the retroviral vector pLXSN to produce pLXSN(U6+1). The H1 promoter region was PCR-amplified from pSilencer 3.0 (Ambion) using the primers 5′-GCCTGCAGGATATTTGCATGTCGCTATGTTCTGG-3′ and 5′-GCTCTAGAGAGTGGTCTCATACAGAACTTATAAG-3′, digested with XbaI and SbfI, and inserted into the pLXSN(U6+1) vector to produce pHybrid.

To test the effectiveness of pHybrid for regulating the expression of an endogenous gene, we constructed a derivative encoding complementary p53-specific sense and antisense RNAs [6]. To this end, the following oligonucleotide was synthesised: 5′-CGGTGATTCCCTCGAGCAAAAAGACTCCAGTGGTAATCTACTTTTTCTAGAGGTAACAGGCGC-3′. Following enzymatic generation of the second strand (as described below), the DNA insert was digested with XhoI and XbaI and ligated between the U6 and H1 convergent promoters in pHybrid to produce pHybrid-p53.

### Enzymatic synthesis of random siRNA insert

A 63 base oligonucleotide template (5′-CGGTGATTCCCTCGAGCAAAAA**N19**TTTTTCTAGAGGTAACAGGCGC-3′) containing 19 random nucleotides (bold) flanked by five adenosines and five thymidines, XhoI and XbaI restriction sites (underlined) and primer binding sites was synthesised and PAGE-purified (International DNA Technologies). A DNA primer (5′-GCGCCTGTTACCTGTAG-3′) was annealed to this template and the complementary strand was synthesised by primer extension using Klenow DNA polymerase. This double-stranded DNA was amplified with PCR primers, 5′-CGGTGATTCCCTCGAGC-3′ and 5′-GCGCCTGTTACCTGTAG-3′. The PCR conditions consisted of 25 cycles of 94°C for 30 sec, 60°C for 30 sec, and 72°C for 45 sec. Purified PCR amplicons were digested with XhoI and ligated to SalI-digested and dephosphorylated pHybrid, with a picomole end ratio of 1∶250 for vector to insert. The ligation products were separated on a 5% Nusieve agarose gel (Cambrex) and the fragment representing the ligation product excised, extracted and purified using a Qiagen gel extraction kit. These ligation products were subsequently digested with XbaI, purified by phenol/chloroform extraction and ethanol precipitation, and subjected to intramolecular ligation to re-circularise all plasmids.

### Library construction

The ligation mixture described above was electroporated into ElectroMAX DH5α-E competent cells (Invitrogen) and the transformed cells plated on 10 cm LB agar plates containing 50 ug/ml carbenicillin. With 2.5 ug ligation mixture, a total of 4.5×10^5^ clones were obtained with 95% of these plasmids containing inserts. DNA sequence analysis of 384 clones indicated the presence of unique inserts and a random distribution of sequences when aligned to the human genome sequence. The library clones were amplified for 48 h at 30°C in 2×LB semi-solid agar containing 50 ug/ml carbenicillin according to the manufacturer's protocol (Invitrogen). Plasmid library stocks were obtained and plasmid DNA prepared using the Qiagen plasmid purification kit.

### Retroviral delivery of pHybrid-p53 or random library and stable cell line production

A total of 4.5×10^6^ Amphopack HEK293 and 6×10^5^ PG13 packaging cells were transfected with 30 ug retroviral-based small RNA library plasmid DNA using Lipofectamine 2000 (Invitrogen) according to the manufacturer's instructions. Medium containing recombinant virus was recovered two days after transfection and 10 ml of virus-containing medium was used to transduce 2.5×10^6^ HCT116 cells for 24 h. Infected cells were selected by adding McCoy's medium, containing 500 ug/ml G418, at 48 h post-transduction. A stable pooled population was obtained eight days post-selection. For pHybrid and pHybrid-p53, the stable pooled populations were serially diluted to isolate single clones. For the random small RNA library, the stable pooled population was subjected to treatment with either 5-FU or a combination of TNF-α and cycloheximide (see below).

### Synthetic siRNA transfection

RNA oligonucleotides composing the siRNAs were annealed according to the manufacturer's specifications (Proligo). The sequences were as follows: Dicer (sense: 5′-UGCUUGAAGCAGCUCUGGA dT dT-3′; antisense: 5′-UCCAGAGCUGCUUCAAGCA dT dT-3′); 5FU55 (sense: 5′-AGCUAAGGAUGCCAGGGAAUU-3′; antisense: 5′-UUCCCUGGCAUCCUUAGCUUU-3′). A non-specific siRNA (sense: 5′- ACUCUAUCUGCACGCUGACUU-3′; antisense: 5′-GUCAGCGAGCAGAUAGAGUUU-3′) was used as a negative control (Dharmacon). Various concentrations of siRNA oligonucleotide duplexes were transfected into 2×10^5^ HCT116 cells alone or HCT116 cells stably transfected with pHybrid or pHybrid-p53 using Lipofectamine 2000 (Invitrogen). At 4 h post-transfection, the transfection mixture was replaced with medium containing 10% FBS, glutamine, penicillin and streptamycin. Cells were harvested 24 and 48 h post-transfection for Western blot analysis.

### Western blot analysis

Pooled populations and stable clones of HCT116 cells, containing pHybrid-p53, were collected at 80% confluency. Cells transfected with synthetic siRNA oligonucleotides were harvested 48 h post-transfection. All collected cell pellets were solubilised and 20 ug of cell lysate separated using 5–12% SDS-PAGE (Novex). Proteins transferred to PVDF membranes (Invitrogen) were probed for the p53 (Oncogene Research) or β-actin (Sigma) proteins, followed by goat HRP-conjugated antibody to mouse IgG (Santa Cruz) or HRP-conjugated antibody to rabbit IgG (Santa Cruz) and detected by chemiluminescence. Immunoblot signal intensity was quantified using ImageQuant software (Molecular Dynamics).

### Genetic selections

A total of 4×10^6^ HCT116 cells containing the random expression library were treated with either 400 uM 5-FU for 18 h or 25 ng/ml TNF-α and 50 ug/ml cycloheximide for 72 h. Cells treated with either 5-FU or TNF-α plus cycloheximide were reseeded at 2×10^5^ cells in T150 cm^2^ flasks and allowed to form colonies for 10–14 days in the absence of 5-FU. Single colonies were isolated and genomic DNA was purified using Qiagen genomic DNeasy tissue kit. Recovery of siRNA expression constructs from genomic DNA was accomplished by PCR with primers 5′- GAACCTCCTCGTTCGACCCCGCCTCGATCC-3′ and 5′-GAGCCTGGGGGACTTTCCACACCCTAACTGAC-3′. The PCR conditions consisted of 40 cycles of 94°C for 1 min, 60°C for 1 min, and 72°C for 1 min. The PCR products were subjected to DNA sequence analysis. To validate inserts from the initial genetic selections, the recovered PCR products were digested with XhoI and XbaI and re-ligated to pHybrid. The recombinant plasmids were purified and re-delivered to HCT116 cells using retroviral transduction. Stable clones were isolated and re-analysed for clonogenic growth over 10–14 days, following pre-treatment with or without 5-FU, as indicated above.

### Assessing 5FU55 siRNA in clonogenic assay

HCT116 cells were seeded at 7×10^4^ cells per well in a 6-well plate. At 24 h after seeding, cells were transfected with 5FU55 siRNA or the non-specific control siRNA (Dharmacon) using two different concentrations and Lipofectamine 2000 according to the manufacturer's instructions. Transfection efficiency was monitored using the siGLO RISC-free siRNA (Dharmacon). At 48 h post-transfection, cells were treated with or without 400 uM 5-FU for 18 h and then reseeded at 2.5×10^4^ cells and allowed to form colonies for 10–14 days. Colonies were visualised and quantified following staining with crystal violet.
